# GC-IMS Combined with Chemometrics Analysis of Volatile Organic Compounds Facilitates CREC Identification

**DOI:** 10.21627/ya61wk37

**Published:** 2026-07-28

**Authors:** Yunwei Zheng, Fuxing Li, Chuwen Zhao, Hong Wang, Shumin Gu, Junqi Zhu, Liuliu Liao, Longhua Hu

**Affiliations:** Jiangxi Province Key Laboratory of Immunology and Inflammation, Jiangxi Provincial Clinical Research Center for Laboratory Medicine, Department of Clinical Laboratory, The Second Affiliated Hospital, Jiangxi Medical College, Nanchang University, Nanchang, Jiangxi, China.; Jiangxi Province Key Laboratory of Immunology and Inflammation, Jiangxi Provincial Clinical Research Center for Laboratory Medicine, Department of Clinical Laboratory, The Second Affiliated Hospital, Jiangxi Medical College, Nanchang University, Nanchang, Jiangxi, China.; Jiangxi Province Key Laboratory of Immunology and Inflammation, Jiangxi Provincial Clinical Research Center for Laboratory Medicine, Department of Clinical Laboratory, The Second Affiliated Hospital, Jiangxi Medical College, Nanchang University, Nanchang, Jiangxi, China.; School of Public Health, Nanchang University, Nanchang, Jiangxi, China.; Department of Laboratory Medicine, Affiliated Hospital of Jiujiang University, Jiujiang Jiangxi 332000, China.; Jiangxi Province Key Laboratory of Immunology and Inflammation, Jiangxi Provincial Clinical Research Center for Laboratory Medicine, Department of Clinical Laboratory, The Second Affiliated Hospital, Jiangxi Medical College, Nanchang University, Nanchang, Jiangxi, China.; Jiangxi Province Key Laboratory of Immunology and Inflammation, Jiangxi Provincial Clinical Research Center for Laboratory Medicine, Department of Clinical Laboratory, The Second Affiliated Hospital, Jiangxi Medical College, Nanchang University, Nanchang, Jiangxi, China.; School of Public Health, Nanchang University, Nanchang, Jiangxi, China.; Department of Urology, Jiangxi Provincial People’s Hospital, The First Affiliated Hospital of Nanchang Medical College, 330000, Nanchang, China.; Jiangxi Province Key Laboratory of Immunology and Inflammation, Jiangxi Provincial Clinical Research Center for Laboratory Medicine, Department of Clinical Laboratory, The Second Affiliated Hospital, Jiangxi Medical College, Nanchang University, Nanchang, Jiangxi, China.

**Keywords:** CREC, GC-IMS, VOCs, IPM, Chemometrics

## Abstract

BACKGROUND: *Escherichia coli* (*E. coli*) is a clinically significant pathogen. Early identification of carbapenem-resistant *E. coli* (CREC) is critical for reducing mortality. METHOD: Gas chromatography-ion mobility spectrometry (GC-IMS) combined with chemometric analysis was used to detect volatile organic compounds (VOCs) from 40 clinical *E. coli* isolates. A two-phase experimental design was implemented: an exploratory phase with six replicates and a validation phase with 40 isolates. Bacteria were cultured in BacT/ALERT® SA broth, and VOCs were analyzed at specific time points. Imipenem (IPM) was added to induce metabolic stress, whereas pyridine-2,6-dicarboxylic acid (DPA) was used to enhance New Delhi metallo-β-lactamase (NDM)-type CREC discrimination. Principal component analysis (PCA) and partial least squares discriminant analysis (PLS-DA) were applied to identify key VOCs. RESULTS: GC-IMS analysis revealed 55 VOCs as detectable signals, of which 30 were tentatively identified. In the absence of IPM, only three VOCs showed significant differences between carbapenem-sensitive *E. coli* (CSEC) and CREC. However, upon the addition of IPM, the number of differentially expressed VOCs increased to 17. PCA and PLS-DA identified 13 key VOCs (including four ketones, two esters, one aldehyde, one pyrazine, and five unknown compounds) that effectively distinguished CSEC from CREC. The addition of DPA further enabled the detection of nine additional VOCs, which may serve as potential markers for identifying NDM-type CREC. CONCLUSION: GC-IMS combined with chemometrics analysis can quickly distinguish CREC from CSEC through analysis of VOC spectra. The addition of IPM and DPA increases metabolic differences, providing a promising method for the rapid detection of CREC.

## Non-Specialist Summary

We investigated whether a gas-sensing method can quickly distinguish ordinary *E. coli* from strains resistant to last-line carbapenem antibiotics. Using 40 clinical samples, we measured tiny airborne chemicals the bacteria release, with and without added drugs that stress them. Changes in a small set of these chemicals allowed us to reliably flag resistant strains, including difficult-to-detect New Delhi metallo-beta-lactamase types, suggesting a practical, rapid lab test.

## 1. Introduction

Carbapenem antibiotics include imipenem (IPM), meropenem, doripenem, and ertapenem. Bacteria are classified as carbapenem-resistant when they exhibit resistance to one or more of these agents. In recent years, the inappropriate use of antibiotics has accelerated the emergence of carbapenem-resistant Enterobacteriaceae (CRE), particularly carbapenem-resistant *Escherichia coli* (CREC), which has substantially complicated clinical treatment strategies [1]. According to the U.S. Centers for Disease Control and Prevention (CDC) Antibiotic Resistance Threats Report, CRE is responsible for thousands of deaths and hundreds of millions of dollars in healthcare expenditures each year in the United States [2]. Data from the China Antimicrobial Surveillance Network (CHINET) indicate that New Delhi metallo-β-lactamase (NDM) remains the most prevalent mechanism of carbapenem resistance among *Escherichia coli* (*E. coli*) isolates. The authors of a multicenter study in China reported that the detection rate of the **bla**NDM gene among CREC isolates was 93% in adults and 97.2% in children [3]. Given its high level of drug resistance and associated mortality, CREC has been designated by the World Health Organization (WHO) as a priority pathogen for which novel antimicrobial therapies are urgently needed [4]. In addition to the development of new therapeutic options, early identification of CREC and implementation of effective infection-control measures in healthcare settings are critical for reducing transmission and mitigating morbidity and mortality among hospitalized patients [5].

Although blood culture combined with antimicrobial susceptibility testing remains the gold standard for pathogen identification, the requirement for overnight incubation limits its utility in urgent clinical settings [6]. Although polymerase chain reaction (PCR) and deoxyribonucleic acid (DNA) sequencing techniques offer high sensitivity and specificity for detecting carbapenemase-encoding genes, their adoption in routine clinical practice is constrained by the need for costly equipment and by relatively high operational expenses [7]. Although various novel diagnostic approaches have emerged in recent years, many face practical barriers such as high technical complexity, limited accessibility, or insufficient validation in real-world clinical environments. As a result, these methods have yet to be fully integrated into routine clinical workflows. Therefore, a critical need remains for innovative, rapid, and cost-effective diagnostic strategies that can be feasibly implemented across diverse healthcare settings.

Microbial volatile organic compounds (mVOCs), which are primary or secondary metabolites generated during bacterial metabolism, naturally volatilize at ambient temperature because of their vapor pressure of at least 1 kPa. These compounds not only reflect microbial growth and metabolic activity but also serve as a chemical fingerprint of the microbial community because distinct bacterial strains generate unique VOC profiles [8]. Accordingly, there has been growing interest in exploring the diagnostic potential of specific VOCs for microbial identification [9].

Recent advancements in analytical chemistry have led to the development of various technologies for VOC detection, including gas chromatography-mass spectrometry (GC-MS) and electronic nose systems. GC-MS provides high sensitivity and access to comprehensive reference databases for VOC identification but requires complex sample pretreatment and lacks real-time detection capability. Electronic noses are portable and suitable for point-of-care applications; however, their low sensitivity and susceptibility to environmental interference limit their clinical utility. By contrast, gas chromatography, on mobility spectrometry (GC-IMS) has demonstrated unique advantages in VOC analysis [10, 11]. This technique does not require complex pretreatment, enables GC-based separation within approximately 10 min, and completes IMS detection within milliseconds, making it particularly suitable for rapid detection. Numerous studies have highlighted the strong potential of GC-IMS for the fast and accurate identification of microorganisms [12, 13].

Given the increasing prevalence of carbapenem resistance in *E. coli* and the urgent need for rapid diagnostic tools, together with findings from our preliminary experiments, we propose the following hypothesis: distinct differences may exist in the VOCs produced by carbapenem-resistant and carbapenem-susceptible *E. coli* strains cultured in the liquid medium of blood culture bottles, and these differences may be further amplified under the selective pressure of carbapenem antibiotics. We further hypothesize that the addition of pyridine-2,6-dicarboxylic acid (DPA) could enhance the detection specificity for strains harboring the **bla**NDM gene. By enabling the rapid identification of CREC—particularly NDM-producing strains—this approach may assist clinicians in making more accurate antibiotic treatment decisions, ultimately improving patient outcomes and helping to curb the spread of carbapenem resistance.

## 2. Materials and Methods

### 2.1. Bacterial Strain Acquisition, Identification, and Sensitivity Testing

The *E. coli* reference strain ATCC 25922 was obtained from the China General Microbiological Culture Collection Center and served as the basic quality control strain for this study. In addition, a total of 40 clinical *E. coli* isolates were collected from the microbiology laboratory of the Second Affiliated Hospital of Nanchang University and included in the experimental cohort. The classification of carbapenem-sensitive *E. coli* (CSEC) and CREC was based on comparisons with the ATCC 25922 control strain and confirmed through species identification using matrix-assisted laser desorption/ionization time-of-flight mass spectrometry (MALDI-TOF/MS). The susceptibility or resistance of these strains to carbapenem antibiotics was further validated using either a VITEK-2 Compact ASTGN16 Automated Antimicrobial Susceptibility Testing System (bioMérieux, Marcy-l’Étoile, France) or the standard Kirby–Bauer disk diffusion method. The minimum inhibitory concentration (MIC) of IPM (Solarbio Science & Technology, Beijing, China) was determined in accordance with the 2022 guidelines of the Clinical and Laboratory Standards Institute (CLSI). To identify the types of carbapenemases produced by CREC isolates, both the modified carbapenem inactivation method (mCIM) and the ethylenediaminetetraacetic acid (EDTA)-enhanced carbapenem inactivation method (eCIM) were performed. In parallel, next-generation sequencing (NGS) technology was used to characterize the antimicrobial resistance genes present in the experimental strains. To ensure consistent viability and stability, all selected CSEC and CREC strains were preserved in brain heart infusion broth supplemented with 15% glycerol (Solarbio Science & Technology, Beijing, China), and stored at –80 °C until required for experimentation, at which point they were thawed and subcultured for use.

### 2.2. Microbiological Culturing Methods

A two-phase experimental design was implemented in this study. The first phase was an exploratory experiment in which one representative CSEC strain and one CREC strain were randomly selected and each tested in six replicates under identical culture conditions. This phase was used to preliminarily assess the potential of VOCs to distinguish CSEC from CREC. The second phase was a validation experiment that included 20 CSEC and CREC strains, with each strain tested in triplicate under the same conditions to establish and validate the VOC-based classification model. All bacterial strains were subcultured onto Columbia blood agar plates the day before experimentation and incubated at 37 °C for 18–22 h to ensure optimal colony formation. On the day of experimentation, a single pure colony was selected and a bacterial suspension with a turbidity equivalent to 0.5 McFarland standard was prepared using sterile normal saline. Nutrient broth from BacT/ALERT® SA (Ref. 259789; bioMérieux, Nürtingen, Germany) served as the enrichment medium. Its components include pancreatic digest of casein (1.7% w/v), papain digest of legume-based food (0.3% w/v), sodium polyanethol sulfonate (0.035% w/v), pyridoxine hydrochloride (0.001% w/v), and mixed amino acids and carbohydrate hydrolysates in purified water [14]. The bacterial suspension was inoculated into test tubes containing the culture medium, and the final bacterial concentration was adjusted to approximately 1 × 10^7^ CFU/mL in a total volume of 6 mL. An equal volume of sterile saline was added to the blank control group. The detailed experimental setup is shown in **Figure S1**.

### 2.3. Antibiotic Treatment

IPM solution was added to a final concentration of 0.25 mg/mL at the T0 time point in some of the experiments to exert metabolic stress and amplify differences between groups [6]. The concentration of the carbapenemase inhibitor, pyridine-2,6-dicarboxylic acid (Solarbio Science & Technology, Beijing, China), was set to 100 mg/L [14, 15]. To avoid degradation of drugs after prolonged placement, all drugs were prepared within 10 min before administration.

### 2.4. GC-IMS Analyses

VOCs in the samples were analyzed using a FlavourSpec® system (Gesellschaft für Analytische Sensorsysteme, G.A.S., Dortmund, Germany), which integrates GC-IMS. The GC unit was equipped with an MXT-WAX capillary column (15 m × 0.53 mm × 1.0 μm; Restek, Bellefonte, PA, USA) and an automatic headspace sampler (CTC-PAL; CTC Analytics AG, Zwingen, Switzerland). The injection needle temperature was maintained at 85 °C, and the column oven temperature was set to 80 °C. Nitrogen gas (purity ≥99.999%) was used as the carrier gas (Beijing Beifen Gas, Beijing, China). The IMS detector featured a drift tube with a length of 98 mm, and nitrogen gas (purity ≥99.999%) was also used as the drift gas at a flow rate of 150 mL/min. All measurements were performed in positive-ion mode. Detailed instrumental parameters are provided in **Supplementary Table S1**, and a schematic of the GC-IMS detection process is shown in **Figure S2**.

### 2.5. Headspace Sampling

The 500 μL sample was placed into a 20 mL headspace glass bottle, sealed with a magnetic screw cap and diaphragm, and shaken at 60 °C and 500 rpm for 3 min. A 1 mL headspace was then automatically absorbed and injected into the instrument. FlavourSpec® was used for the analysis. The retention index (RI) of each VOC was determined using C4–C9 *n*-ketones standards (Sinopharm Chemical Reagent Beijing, Beijing, China) under identical analytical conditions, with a tolerance window of ± 50% RI units applied during matching. For precise identification, both the RI and drift time (Dt) of the monomer and dimer ions were simultaneously compared to reference data in the National Institute of Standards and Technology (NIST, Gaithersburg, MD, United States) 2014 library and in the GC-IMS database provided by G.A.S. (Dortmund, Germany). A compound was assigned only when both the RI and Dt values matched those in the reference databases within the defined tolerances ( ± 50% RI units; ± 2% for Dt), and the characteristic ion pattern (e.g., monomer/dimer ratio) was consistent. This approach enabled high-confidence annotation of the detected VOCs on the basis of multi-parameter matching. However, in the absence of authentic standard verification, all identifications are considered tentative and should be interpreted as putative assignments.

### 2.6. GC-IMS Data Analyses

GC-IMS data were analyzed using VOCal Version 0.1.3, the FlavourSpec® software package. The VOCal plug-in Galerie was used to map fingerprints, the Gallery Plot plug-in was used to compare fingerprints between groups, and Nearest Neighbour was used to perform similarity analysis.

### 2.7. Statistical Methods

Bacterial growth curves were generated using GraphPad Prism 8.3.0 (GraphPad Software, San Diego, CA, USA). The Mann–Whitney U-test was used to assess differences in VOC profiles between the two groups, and the resultant *p*-values were adjusted to control the false discovery rate (FDR). Differences were considered statistically significant when FDR < 0.05. Principal component analysis (PCA) and partial least squares discriminant analysis (PLS-DA) were performed using MetaboAnalyst 5.0, an online analytical platform (https://www.metaboanalyst.ca). Prior to multivariate analysis, the data were log-transformed (base 10) to reduce skewness. Potential discriminatory VOCs were selected on the basis of a variable importance in projection (VIP) score > 1 and FDR < 0.05. A classification model for CREC identification was subsequently developed. Model stability and predictive performance were evaluated through cross-validation (*R*^2^ and *Q*^2^ values) and 1000 permutation tests.

## 3. Results

### 3.1. Analysis of Drug Resistance and Enzyme Types

The results of the drug sensitivity of 20 strains of *E. coli* to four carbapenem antibiotics are shown in **Supplementary Table S2**; none of the strains showed simultaneous sensitivity or intermediate resistance to all four antibiotics.

### 3.2. Growth Curve of ATCC-25922

The growth curve of *E. coli* (ATCC 25922) in the liquid medium for blood culture bottles is shown in **Figure 1A**. The optical density (OD) value was measured every hour. The bacteria entered the exponential growth phase after 2 h and reached the end of this stage approximately 5 h after inoculation. On the basis of this growth pattern, five time points were selected during the 3–7 h proliferation period for VOC analysis: 3 h (T0), 4 h (T1), 5 h (T2), 6 h (T3), and 7 h (T4). The T2 time point, corresponding to the end of the exponential growth phase, was chosen for comparing VOC differences between groups.

**Figure 1.**
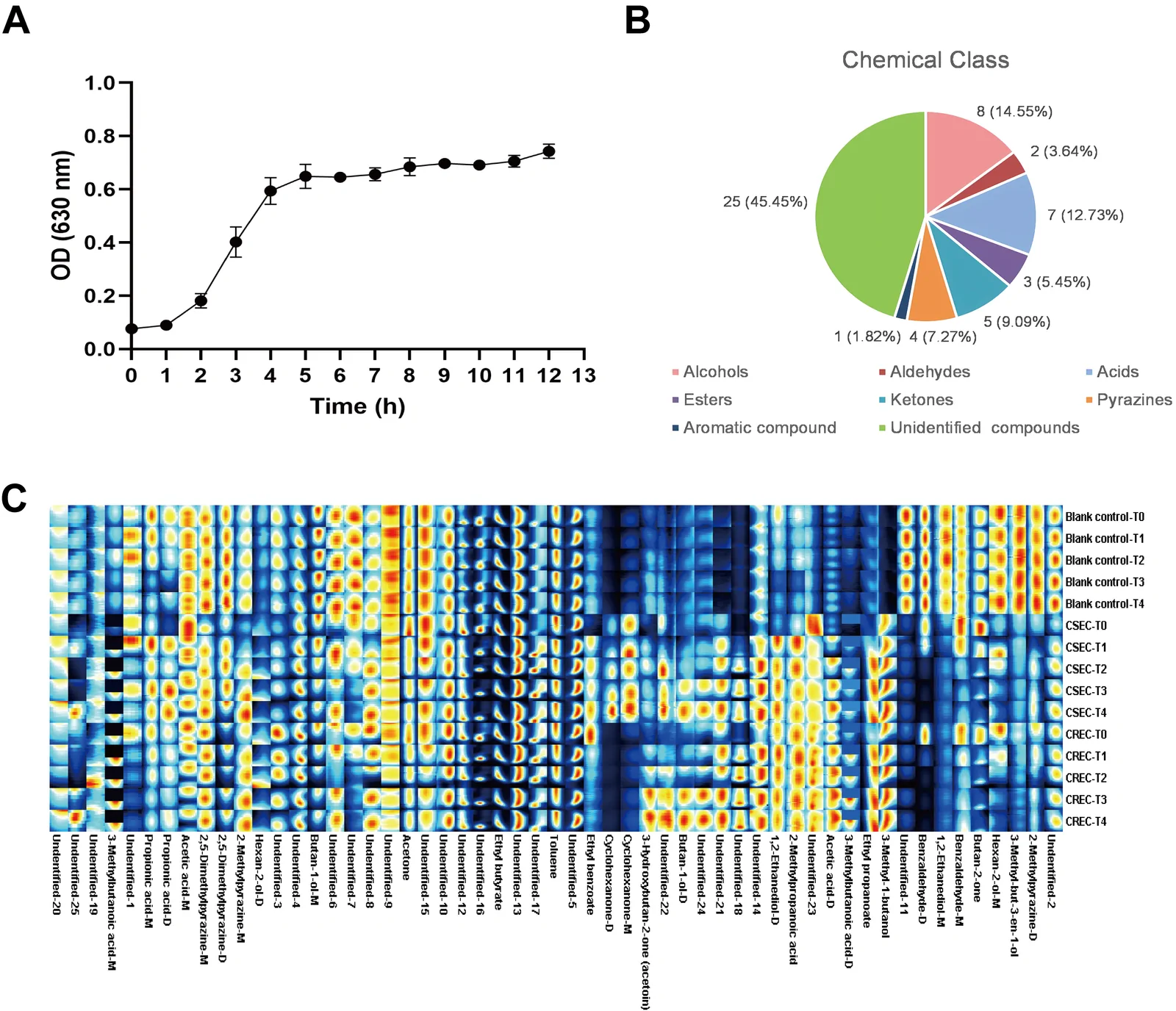
(A) Growth curve of *Escherichia coli* (ACTT-25922). (B) Types, quantities, and percentages of 55 VOCs. (C) Fingerprints of blank bottles, CSEC, and CREC generated at time points T0–T4.

### 3.3. Metabolite Analysis at Time Points T0–T4

A total of 55 VOCs were detected by GC-IMS, of which 30 were tentatively identified by matching retention indices, drift times, and molecular weights against reference databases. The CAS Registry Numbers of the various compounds, along with their retention indices, retention times, drift times in the electric field, and other detailed information, are presented in **Supplementary Table S3**. These compounds include eight alcohols, seven acids, five ketones, four pyrazines, three esters, two aldehydes, one aromatic compound, and 25 unknown compounds (**Figure 1B**). The fingerprints generated by the blank bottle, CSEC, and CREC in the medium of aerobic blood culture flasks showed no difference in type, except for the differences in signal intensity (**Figure 1C**). The CSEC and CREC groups showed no significant differences in their fingerprints.

### 3.4. Exploring VOC Discrimination at Point T2 Before IPM Addition

At the T2 time point, we compared the differences in VOC profiles between the CSEC and CREC groups and separately with the blank control group (see **Supplementary Table S4** for the detailed T0–T4 data).

Compared with the blank control group, 26 VOCs showed increased levels and 29 VOCs exhibited decreased levels in the CSEC group. In the CREC group, 24 VOCs were upregulated and 31 were downregulated. A total of 49 VOCs displayed consistent trends in both the CSEC and CREC groups, including 22 upregulated and 27 downregulated compounds. Only six VOCs demonstrated opposing trends between the two groups: 1,2-ethanediol-M, propionic acid-M, 3-methylbutanoic acid-M, ethyl benzoate, cyclohexanone-D, and 2,5-dimethylpyrazine-D. Notably, although only seven VOCs in the CSEC group differed significantly from the blank control, as many as 37 VOCs in the CREC group showed significant differences compared with the control. However, only three statistically significant differences were observed when comparing CSEC and CREC directly: two acidic compounds (acetic acid-D and 2-methylpropanoic acid) and one ester compound (ethyl benzoate), as summarized in **Table 1**. The volatile profiles of all 55 VOCs across different groups at time point T2 are presented in **Figure 2A**. Visual inspection of the fingerprint patterns revealed no distinct separation between CSEC and CREC strains on the basis of their carbapenem susceptibility profiles.

**Figure 2.**
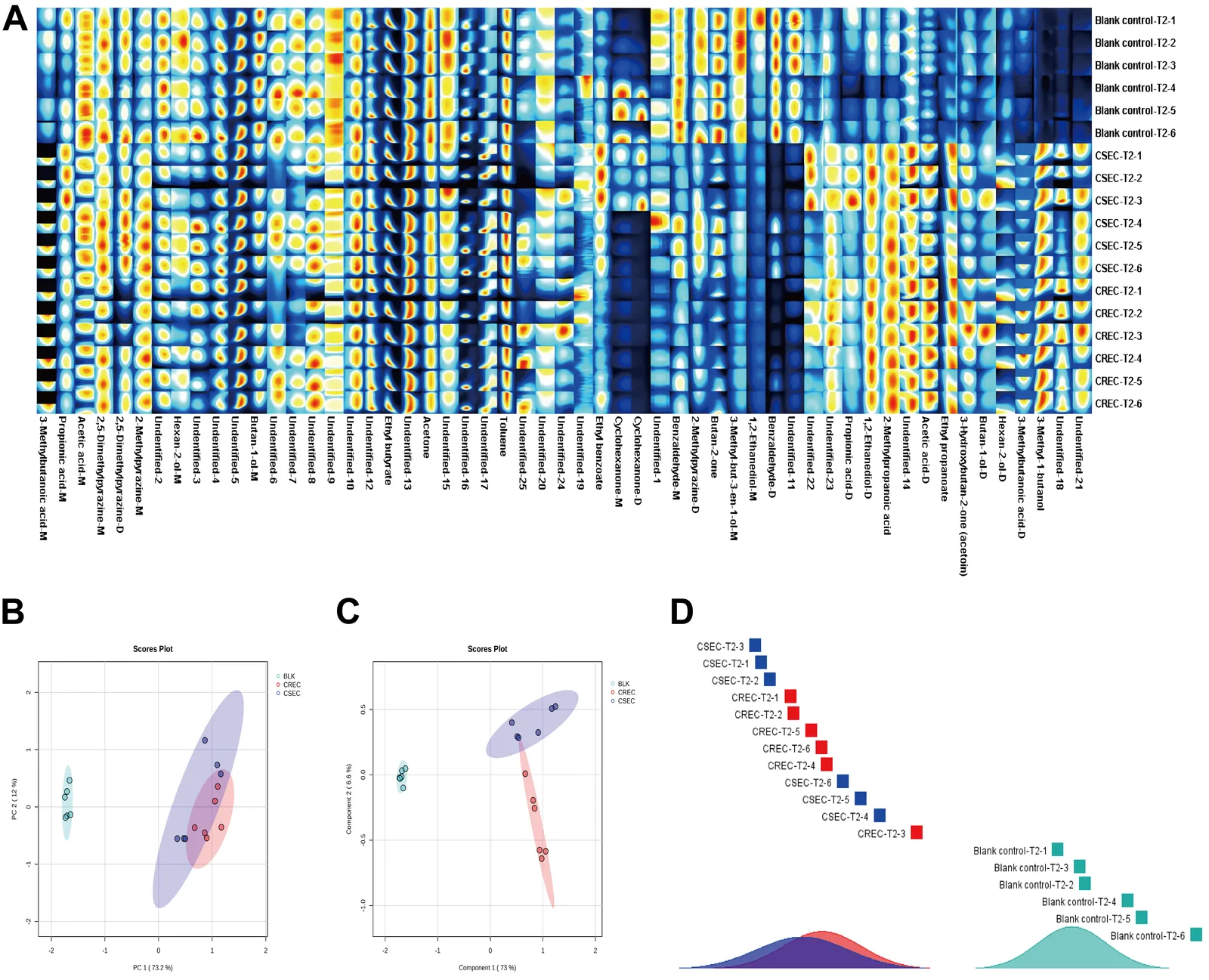
(A) Fingerprints of blank bottles, CSEC, and CREC generated at time point T2. (B) PCA analysis of three groups without IPM at time point T2. (C) PLS-DA analysis of three groups without IPM at T2 time points. (D) Similarity analysis of three groups without IPM at time point T2.

**Table 1.** With reference to the blank bottle, the comparative change trend of VOCs relative content between CSEC and CREC at time point T2.

Label	Blank control mean ± SD (*n* = 6)	CSEC mean ± SD (*n* = 6)	Up/Down	CREC mean ± SD (*n* = 6)	Up/Down	FDR_1_	FDR_2_	FDR_3_
1,2-Ethanediol-D	363.76 ± 31.32	304.71 ± 22.85	D	325.21 ± 20.58	D	0.1697	0.0440	0.4321
1,2-Ethanediol-M	82.24 ± 3.62	84.38 ± 5.24	U	79.84 ± 9.28	D	0.8681	0.5783	0.7070
3-Methyl-but-3-en-1-ol	347.45 ± 17.21	229.21 ± 34.03	D	213.70 ± 27.34	D	0.8425	5.131E-06	0.8245
Butan-1-ol-D	105.71 ± 24.67	160.96 ± 60.99	U	170.48 ± 42.05	U	0.8773	0.0159	0.9283
Butan-1-ol-M	595.46 ± 60.05	560.01 ± 79.72	D	531.75 ± 108.52	D	0.2607	0.2772	0.8501
Hexan-2-ol-D	58.25 ± 11.38	206.22 ± 53.43	U	235.69 ± 53.90	U	0.2612	4.2983E-05	0.7700
Hexan-2-ol-M	164.17 ± 23.72	110.81 ± 17.18	D	90.61 ± 24.00	D	0.2311	0.0009	0.4282
3-Methyl-1-butanol	318.14 ± 33.13	1342.81 ± 143.5	U	1359.24 ± 109.83	U	0.3034	1.0476E-08	0.9297
Benzaldehyde-D	3588.22 ± 47.01	899.65 ± 618.31	D	405.29 ± 247.09	D	0.1679	7.8805E-10	0.3630
Benzaldehyde-M	1137.32 ± 32.73	756.53 ± 198.29	D	657.92 ± 75.39	D	0.3031	3.0663E-07	0.7371
Acetic acid-D	1455.06 ± 44.87	3327.24 ± 417.67	U	4445.77 ± 159.37	U	0.4608	4.5978E-11	0.0061
Acetic acid-M	1833.32 ± 172.45	1556.31 ± 185.81	D	1576.60 ± 112.79	D	0.8692	0.0210	0.9639
Propionic acid-D	65.89 ± 19.25	148.49 ± 34.74	U	121.14 ± 23.91	U	0.8732	0.0032	0.4381
Propionic acid-M	685.38 ± 89.62	741.51 ± 113.14	U	580.08 ± 57.96	D	0.0412	0.0511	0.1216
2-Methylpropanoic acid	379.56 ± 33.95	257.43 ± 10.45	D	285.09 ± 9.47	D	0.3078	0.0002	0.0132
3-Methylbutanoic acid-D	178.51 ± 23.50	186.62 ± 17.63	U	218.39 ± 15.42	U	0.6957	0.0117	0.1060
3-Methylbutanoic acid-M	458.07 ± 30.15	406.24 ± 49.38	D	482.87 ± 43.48	U	0.6829	0.3116	0.1180
Ethyl propanoate	311.05 ± 50.16	913.32 ± 126.72	U	980.27 ± 92.87	U	0.2653	1.5294E-07	0.7360
Ethyl butyrate	3061.49 ± 142.39	2887.26 ± 228.73	D	2883.88 ± 89.25	D	0.3803	0.0401	0.9937
Ethyl benzoate	346.37 ± 9.23	727.95 ± 102.44	D	465.93 ± 77.27	U	0.8796	0.0081	0.0147
Acetone	6032.86 ± 257.84	5267.23 ± 614.81	D	5375.10 ± 360.49	D	0.5525	0.0097	0.9191
Butan-2-one	4106.45 ± 311.49	2242.55 ± 474.62	D	1708.21 ± 91.09	D	0.8104	5.2054E-08	0.1344
Cyclohexanone-D	75.35 ± 2.46	314.02 ± 270.52	U	67.85 ± 5.43	D	0.8822	0.0206	0.2290
Cyclohexanone-M	24.62 ± 2.86	77.13 ± 39.33	U	35.66 ± 6.93	U	0.2814	0.0097	0.1606
3-Hydroxybutan-2-one (acetoin)	60.17 ± 9.23	114.40 ± 23.17	U	125.90 ± 10.53	U	0.8644	1.9983E-06	0.7357
2-Methylpyrazine-D	229.75 ± 60.18	196.85 ± 46.42	D	153.22 ± 33.97	D	0.5523	0.0333	0.3647
2-Methylpyrazine-M	283.32 ± 2.78	313.63 ± 36.48	U	303.64 ± 30.33	U	0.8690	0.1666	0.8703
2,5-Dimethylpyrazine-D	359.02 ± 59.19	405.59 ± 53.36	U	270.20 ± 97.8	D	0.3861	0.1102	0.1091
2,5-Dimethylpyrazine-M	1055.95 ± 112.87	957.17 ± 171.76	D	913.11 ± 49.49	D	0.1189	0.0293	0.8790
Toluene	5607.4 ± 258.63	5244.20 ± 437.31	D	5414.43 ± 358.35	D	0.0464	0.3343	0.8211
unidentified-1	78.26 ± 9.77	43.84 ± 10.30	D	33.91 ± 4.76	D	0.3009	5.4871E-06	0.2443
unidentified-2	1139.52 ± 53.25	974.35 ± 53.33	D	988.00 ± 63.84	D	0.2869	0.0030	0.9118
unidentified-3	207.36 ± 20.00	568.10 ± 144.8	U	547.56 ± 142.55	U	0.3962	0.0005	0.9678
unidentified-4	1312.17 ± 59.38	1846.42 ± 112.40	U	1813.29 ± 91.16	U	0.2773	2.2018E-06	0.8500
unidentified-5	6013.76 ± 157.35	5455.78 ± 328.95	D	5495.65 ± 287.28	D	0.2892	0.0071	0.9483
unidentified-6	40.64 ± 12.60	47.46 ± 20.53	U	46.88 ± 27.81	U	0.3656	0.6272	0.9937
unidentified-7	83.83 ± 10.62	76.06 ± 14.16	D	62.99 ± 15.51	D	0.3038	0.0341	0.4578
unidentified-8	164.9 ± 11.15	128.62 ± 15.54	D	122.96 ± 18.50	D	0.0464	0.0020	0.8849
unidentified-9	268.25 ± 47.87	220.55 ± 83.95	D	226.1 ± 107.98	D	0.2835	0.4178	0.9937
unidentified-10	2333.23 ± 142.06	2264.84 ± 202.81	D	2172.56 ± 208.67	D	0.4256	0.1794	0.8346
unidentified-11	293.33 ± 35.88	366.36 ± 36.49	U	345.66 ± 29.17	U	0.2738	0.0319	0.7251
unidentified-12	1938.88 ± 105.02	1894.94 ± 101.70	D	1864.06 ± 139.57	D	0.0269	0.3371	0.8997
unidentified-13	7713.76 ± 280.60	7398.11 ± 693.13	D	7501.36 ± 338.53	D	0.2722	0.3026	0.9372
unidentified-14	1496.09 ± 98.58	3323.25 ± 454.32	U	3485.78 ± 252.06	U	0.2737	4.6929E-08	0.8184
unidentified-15	701.32 ± 30.88	549.22 ± 71.67	D	460.91 ± 47.23	D	0.5610	4.2152E-06	0.1518
unidentified-16	5798.26 ± 429.00	1604.53 ± 513.13	D	1468.97 ± 271.59	D	0.0395	1.5448E-08	0.8621
unidentified-17	337.11 ± 15.56	1082.62 ± 120.80	U	1082.06 ± 72.61	U	0.8653	5.2153E-09	0.9937
unidentified-18	108.90 ± 13.94	1750.18 ± 488.50	U	2521.86 ± 369.84	U	0.3875	1.3145E-07	0.1058
unidentified-19	24.66 ± 2.91	21.67 ± 0.89	D	19.72 ± 4.08	D	0.2735	0.0502	0.7688
unidentified-20	34.43 ± 3.67	43.26 ± 4.18	U	43.45 ± 5.51	U	0.2356	0.0142	0.9937
unidentified-21	15.17 ± 2.99	182.03 ± 31.31	U	182.19 ± 33.46	U	0.2718	1.2722E-06	0.9937
unidentified-22	58.60 ± 3.31	63.72 ± 25.82	U	71.26 ± 14.51	U	0.2965	0.0834	0.8853
unidentified-23	552.39 ± 27.39	457.28 ± 28.93	D	483.87 ± 72.58	D	0.8545	0.0748	0.8329
unidentified-24	108.27 ± 19.66	114.79 ± 16.85	U	124.43 ± 29.85	U	0.0017	0.3237	0.8449
unidentified-25	77.76 ± 50.89	101.93 ± 74.29	U	139.35 ± 81.8	U	0.0060	0.1814	0.8084

Note: FDR_1_: CSEC vs Blank control; FDR_2_: CREC vs Blank control; FDR_3_: CSEC vs CREC.

Abbreviations: VOCs, volatile organic compounds; CSEC, Carbapenem-sensitive *Escherichia coli*; CREC, Carbapenem-resistant *Escherichia coli*; FDR, false discovery rate; M, monomer; D, dimer; SD, standard deviation.

PCA further showed that the first principal component explained 73.2% of the total variance and the second principal component explained 12%, with a cumulative contribution of 85.2%. The PCA score plot (**Figure 2B**) shows a clear separation between the blank control group and the other two groups; however, the samples were more clustered within the CSEC and CREC groups, which could not be effectively differentiated. The PLS-DA results (**Figure 2C**) also show that the blank control group could still be clearly distinguished, whereas some overlap was observed between the CSEC and CREC groups. The results of similarity analysis were consistent with PCA and PLS-DA, indicating that effective differentiation between CSEC and CREC groups could not yet be achieved by VOC mapping without the addition of IPM (**Figure 2D**).

### 3.5. Effect of Adding IPM on VOC Generation

To amplify the potential metabolic differences between CSEC and CREC, we added IPM to the culture system at the T0 time point to exert metabolic stress and further analyze its effect on VOC production.

With the addition of IPM, no novel VOCs were detected in any of the three groups during the T0–T4 periods (see **Supplementary Table S5** for detailed temporal data). **Table 2** presents the relative changes in VOC levels across groups at time point T2. In the presence of IPM, 25 VOCs showed increased levels and 30 exhibited decreased levels in the CSEC group. In the CREC group, 24 VOCs were upregulated and 31 were downregulated. Among these, 42 VOCs displayed consistent trends in both the CSEC and CREC groups: 23 were downregulated and 19 were upregulated. The number of VOCs showing opposing trends increased to 13: 1,2-ethanediol-D, 1,2-ethanediol-M, butan-1-ol-D, 3-methylbutanoic acid-D, 3-methylbutanoic acid-M, ethyl benzoate, cyclohexanone-D, 2-methylpyrazine-M, 2,5-dimethylpyrazine-D, and unidentified-6, -7, -8, and -23. Notably, the number of statistically distinct VOCs between CSEC and CREC increased from three in the absence of IPM to 17 in its presence. Visual comparison of the fingerprint profiles in **Figure 2A** and **Figure 3A** revealed a marked reduction in the ion current signal intensity of 12 VOCs (i.e., 3-methylbutanoic acid-D, 3-methyl-1-butanol, ethyl propanoate, acetic acid-D, 1,2-ethanediol-D, 3-hydroxybutan-2-one (acetoin), 2-methylpropanoic acid, and unidentified-14, -18, -21, -22, and -23) compared with their signal intensities in the absence of IPM. This decrease in signal intensity was observed even though not all of these substances showed statistically significant differences in the quantitative analysis between CSEC and CREC.

**Figure 3.**
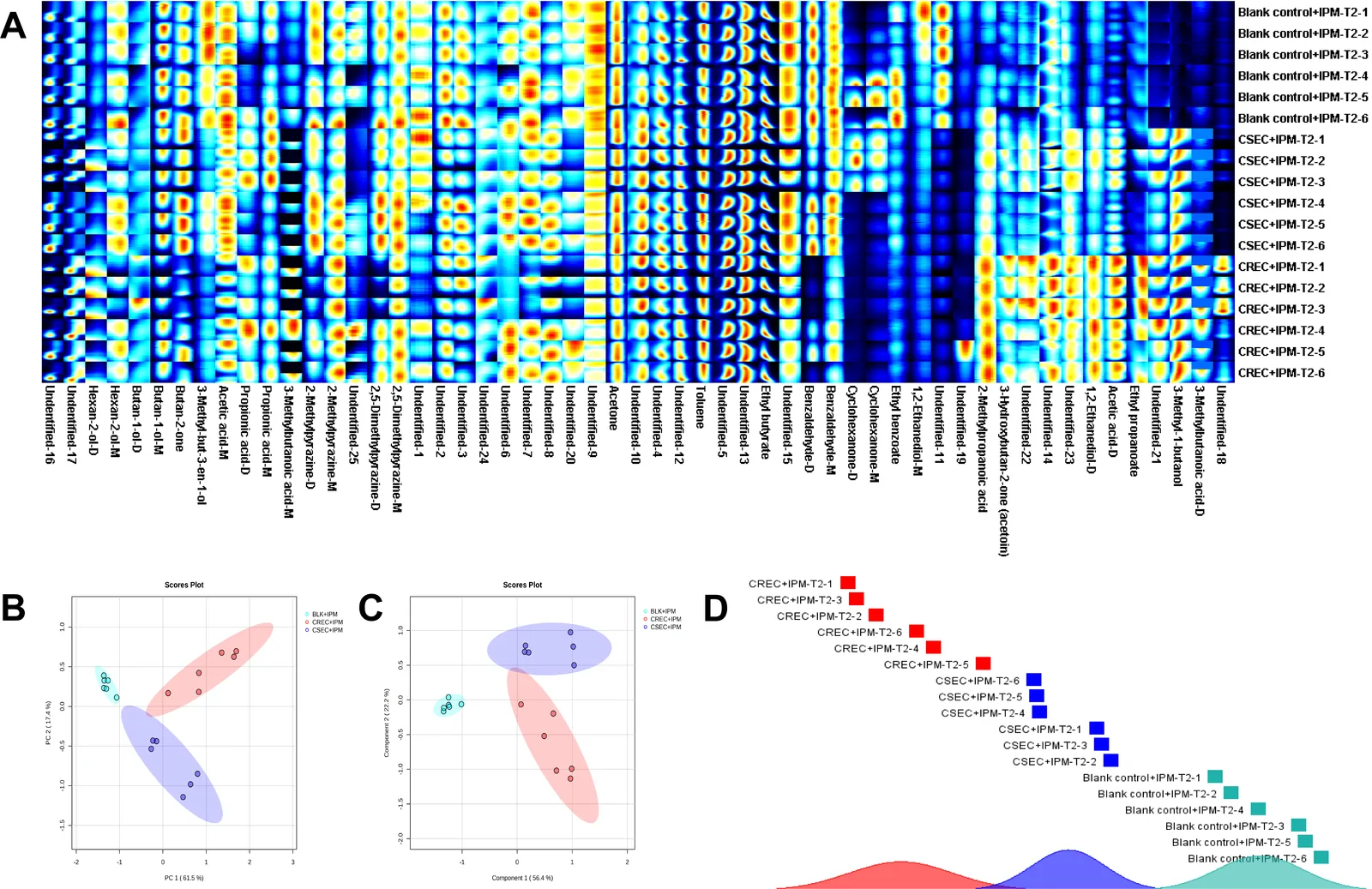
(A) Fingerprints of blank bottles+IPM, CSEC+IPM, and CREC+IPM generated at time point T2. (B) PCA analysis was performed on the three groups at time point T2 after IPM was added. (C) PLS-DA analysis was performed on the three groups at time point T2 after IPM was added. (D) After IPM was added, the similarity analysis of the three groups was performed at time point T2.

**Table 2.** Relative VOC content at time point T2 after IPM was added to blank bottles, CSEC, and CREC.

Label	Blank control + IPM mean ± SD (*n* = 6)	CSEC + IPM mean ± SD (*n* = 6)	Up/Down	CREC + IPM mean ± SD (*n* = 6)	Up/Down	FDR_1_	FDR_2_	FDR_3_
1,2-Ethanediol-D	264.32 ± 21.99	256.32 ± 47.98	D	333.48 ± 34.17	U	0.7453	0.0042	0.0303
3-Methyl-but-3-en-1-ol	342.06 ± 19.79	262.47 ± 28.52	D	218.27 ± 32.88	D	0.0011	7.2107E-05	0.0707
Hexan-2-ol-D	101.92 ± 3.84	198.33 ± 47.30	U	239.97 ± 67.64	U	0.0020	0.0014	0.3847
Hexan-2-ol-M	166.96 ± 14.36	115.69 ± 22.62	D	94.23 ± 22.10	D	0.0028	0.0002	0.2261
3-Methyl-1-butanol	222.38 ± 9.21	1247.6 ± 72.64	U	1299.60 ± 180.76	U	5.7833E-10	8.4246E-07	0.6754
Benzaldehyde-D	3387.53 ± 78.16	2350.17 ± 465.03	D	825.11 ± 750.46	D	0.0013	5.1025E-05	0.0120
Benzaldehyde-M	1222.53 ± 30.54	1029.32 ± 102.78	D	692.48 ± 126.97	D	0.0038	1.3175E-05	0.0055
Acetic acid-D	1388.35 ± 125.92	2827.27 ± 522.93	U	4332.05 ± 358.77	U	0.0007	1.9843E-07	0.0023
Acetic acid-M	1865.39 ± 58.01	1373.15 ± 209.91	D	1472.52 ± 62.67	D	0.0011	5.7914E-06	0.4234
Propionic acid-D	33.86 ± 10.14	119.55 ± 30.70	U	129.40 ± 26.19	U	0.0005	5.6507E-05	0.6884
2-Methylpropanoic acid	297.40 ± 28.66	215.35 ± 11.61	D	289.08 ± 28.58	D	0.0006	0.6619	0.0029
3-Methylbutanoic acid-D	164.71 ± 15.13	147.53 ± 7.06	D	231.03 ± 44.66	U	0.0642	0.0128	0.0087
Ethyl propanoate	331.89 ± 37.41	411.83 ± 105.13	U	881.93 ± 215.56	U	0.1548	0.0003	0.0066
Ethyl benzoate	477.57 ± 12.38	550.36 ± 41.04	U	429.71 ± 60.42	D	0.0054	0.1322	0.0129
Acetone	6182.56 ± 104.82	5837.35 ± 713.95	D	5547.27 ± 550.37	D	0.3355	0.0347	0.5878
Butan-2-one	3936.19 ± 370.00	3781.48 ± 214.76	D	2250.25 ± 586.02	D	0.4846	0.0004	0.0036
Cyclohexanone-D	79.83 ± 2.82	308.77 ± 245.56	U	64.82 ± 6.08	D	0.0834	0.0008	0.0747
Cyclohexanone-M	26.50 ± 3.18	90.50 ± 44.91	U	38.63 ± 6.53	U	0.0148	0.0046	0.0575
3-Hydroxybutan-2-one (acetoin)	53.15 ± 9.94	81.62 ± 12.20	U	119.71 ± 20.80	U	0.0039	0.0002	0.0143
2-Methylpyrazine-D	303.02 ± 25.05	250.71 ± 46.02	D	159.43 ± 50.06	D	0.0678	0.0004	0.0281
2-Methylpyrazine-M	277.80 ± 11.31	255.16 ± 14.86	D	280.79 ± 22.78	U	0.0322	0.7934	0.0888
2,5-Dimethylpyrazine-M	1118.39 ± 88.22	941.73 ± 209.81	D	892.6 ± 27.48	D	0.1321	0.0005	0.6959
unidentified-1	57.96 ± 5.74	49.77 ± 8.89	D	36.99 ± 10.69	D	0.1297	0.0040	0.0941
unidentified-2	1137.48 ± 58.96	942.21 ± 75.97	D	953.84 ± 65.48	D	0.0019	0.0013	0.8274
unidentified-3	490.11 ± 41.7	606.24 ± 96.81	U	568 ± 143.81	U	0.0492	0.2767	0.7031
unidentified-4	1698.16 ± 30.28	1876.27 ± 80.75	U	1827.21 ± 90.45	U	0.0019	0.0154	0.4628
unidentified-7	67.79 ± 4.23	75.18 ± 8.45	U	55.67 ± 9.87	D	0.1323	0.0343	0.0167
unidentified-10	1689.30 ± 44.34	2141.33 ± 61.85	U	1859.75 ± 161.34	U	1.2891E-06	0.0528	0.0128
unidentified-11	297.98 ± 18.74	386.82 ± 32.50	U	325.33 ± 32.14	U	0.0010	0.1515	0.0296
unidentified-14	1481.22 ± 102.76	1974.18 ± 549.11	U	3262.04 ± 610.47	U	0.0933	0.0001	0.0138
unidentified-15	656.03 ± 41.80	566.62 ± 43.34	D	479.44 ± 65.22	D	0.0119	0.0007	0.0586
unidentified-16	5170.63 ± 360.47	2575.73 ± 996.78	D	1829.14 ± 706.94	D	0.0009	1.0962E-05	0.2757
unidentified-17	490.46 ± 36.67	639.76 ± 145.21	U	1095.82 ± 92.17	U	0.0659	9.9382E-07	0.0038
unidentified-18	135.98 ± 21.34	286.27 ± 200.05	U	1822.22 ± 880.44	U	0.1407	0.0021	0.0118
unidentified-19	302.53 ± 83.81	51.64 ± 23.41	D	104.80 ± 124.26	D	0.0005	0.0171	0.4617
unidentified-20	158.05 ± 15.55	84.61 ± 26.60	D	105.29 ± 42.63	D	0.0010	0.0317	0.4634
unidentified-21	21.94 ± 5.64	139.02 ± 22.05	U	179.49 ± 31.64	U	3.3823E-06	3.9957E-06	0.0667
unidentified-22	50.36 ± 5.90	59.83 ± 7.08	U	77.93 ± 14.33	U	0.0621	0.0034	0.0568
unidentified-24	172.57 ± 23.4	134.56 ± 13.15	D	150.05 ± 30.85	D	0.0144	0.2421	0.4225

Note: FDR_1_: CSEC + IPM vs Blank control + IPM; FDR_2_: CREC + IPM vs Blank control + IPM; FDR_3_: CSEC + IPM vs CREC + IPM.

Abbreviations: VOC, volatile organic compound; CSEC, Carbapenem-sensitive *Escherichia coli*; CREC, Carbapenem-resistant Escherichia coli; IPM, Imipenem; FDR, false discovery rate; M, monomer; D, dimer; SD, standard deviation.

A classification model distinguishing CSEC and CREC was reconstructed using multivariate statistical analysis. PCA revealed that the first principal component explained 61.5% of the total variance, whereas the second principal component accounted for 17.4%, resulting in a cumulative contribution rate of 79.1% (**Figure 3B**). Although this cumulative variance was lower than that of the model without IPM, PLS-DA analysis (**Figure 3C**) revealed nearly complete separation between the CSEC and CREC groups. Furthermore, similarity analysis (**Figure 3D**) demonstrated clear differentiation among all three experimental groups.

The cross-validation results (**Figure S3A**) demonstrated *R*^2^ and *Q*^2^ values of 0.99252 and 0.93262, respectively (with *Q*^2^ > 0.4 indicating acceptable discriminative capacity). The outcomes of 1000 permutation tests (**Figure S3B**) revealed no evidence of overfitting (*p* = 0.017), suggesting that the model exhibited high stability and strong predictive performance.

### 3.6. Key VOCs Distinguishing CSEC from CREC

Following the introduction of IPM and optimization of the experimental conditions, we developed a chemometric classification model to distinguish CSEC from CREC. Notably, with an increased sample size, PCA (**Figure 4A**) and PLS-DA (**Figure 4B**) demonstrated effective separation between the CSEC+IPM and CREC+IPM groups. Cross-validation results (**Figure S3C**) revealed *R*^2^ and *Q*^2^ values of 0.98033 and 0.97020, respectively. In addition, 1000 permutation tests (**Figure S3D**) yielded a *p*-value < 0.001, indicating that the model exhibited high stability and strong predictive performance. VIP values for all 55 VOCs were calculated, and those with VIP > 1 and FDR < 0.05 were selected as statistically significant features. As illustrated in **Figure S4A**, VOCs were ranked by descending VIP values, identifying 13 compounds with VIP > 1. Further analysis revealed that 48 VOCs had FDR < 0.05 (**Table 3**). Among them, 13 VOCs met both selection criteria: five unidentified compounds, four ketones, two esters, one aldehyde, and one pyrazine derivative. Of these 13 differentially expressed VOCs, unidentified-18, unidentified-11, ethyl propanoate, unidentified-19, unidentified-17, 3-hydroxybutan-2-one (acetoin), and ethyl benzoate showed significantly higher levels in the CREC+IPM group than in the CSEC+IPM group. By contrast, benzaldehyde-D, cyclohexanone-D, cyclohexanone-M, unidentified-10, butan-2-one, and 2-methylpyrazine-D exhibited inverse trends (**Figure S4B**). The relative abundance of these 13 discriminatory VOCs across the CSEC+IPM and CREC+IPM groups is visualized as a heatmap (**Figure 4C**), clearly illustrating substantial differentiation between the two groups.

**Figure 4.**
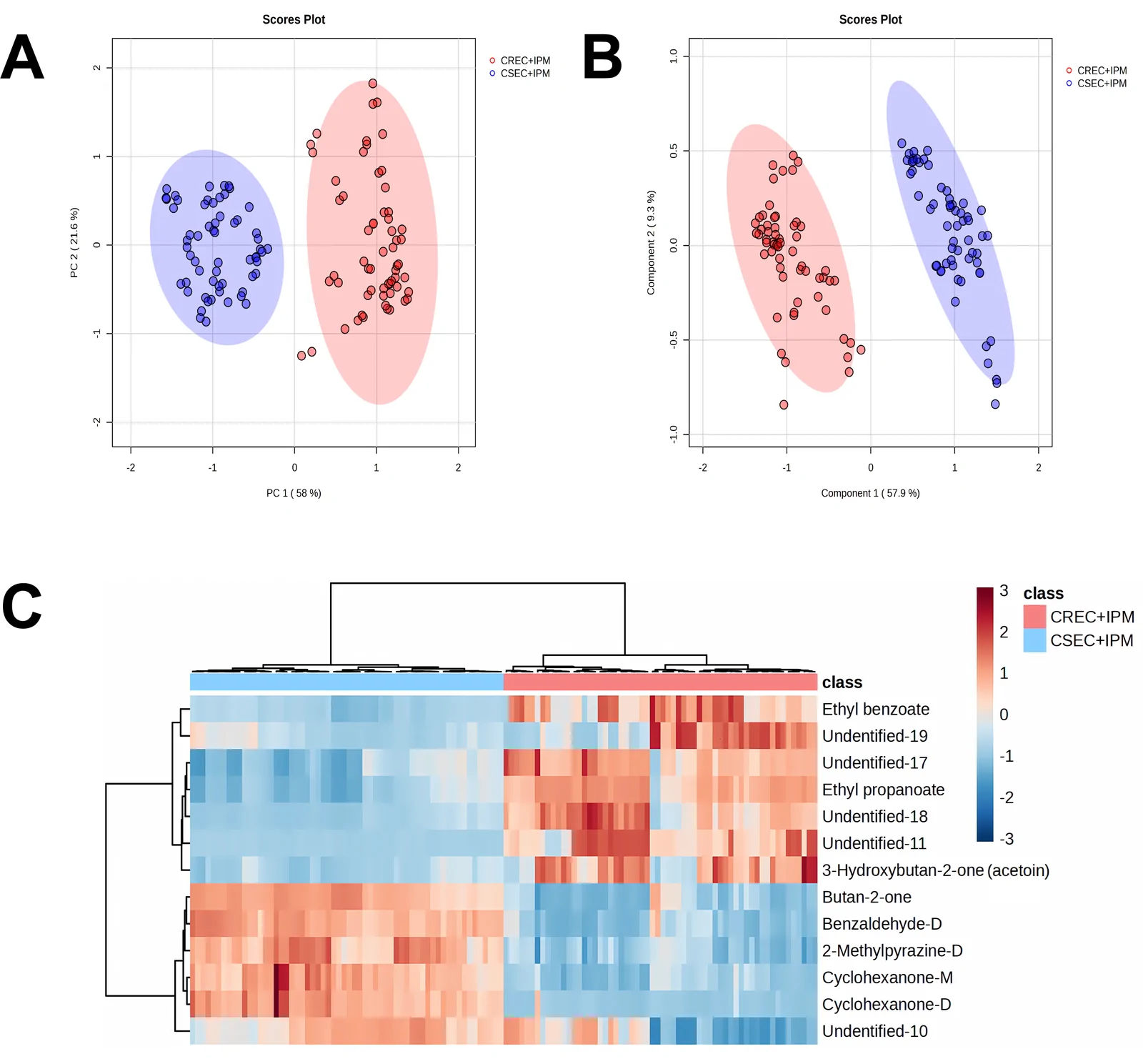
(A) PCA analysis of 60 parallel experiments at time point T2. (B) PLS-DA analysis of 60 parallel experiments at time point T2. (C) The relative abundance of 13 different VOCs in the two groups.

**Table 3.** Comparison of VOC content at time point T2 between 20 strains of CREC and CSEC strains after IPM addition.

Label	CSEC + IPM (T2) mean ± SD (*n* = 60)	CREC + IPM (T2) mean ± SD (*n* = 60)	VIP	*p*	FDR
unidentified-18	224.12 ± 164.60	1639.15 ± 645.16	3.63	2.3782E-32	1.1891E-31
unidentified-11	41.13 ± 4.99	239.50 ± 94.22	2.85	5.7903E-32	2.6539E-31
Benzaldehyde-D	2945.75 ± 354.62	735.15 ± 553.25	2.73	9.0179E-51	2.4799E-49
Cyclohexanone-D	199.07 ± 31.80	65.64 ± 16.96	1.89	5.1176E-55	2.8147E-53
Ethyl propanoate	368.05 ± 136.15	944.20 ± 145.45	1.68	2.5620E-44	4.6969E-43
unidentified-19	233.06 ± 181.61	772.59 ± 563.71	1.68	1.2577E-10	2.0961E-10
Cyclohexanone-M	50.41 ± 8.29	22.66 ± 5.26	1.37	2.3350E-43	3.2106E-42
unidentified-17	496.64 ± 172.78	1042.20 ± 179.41	1.33	1.9304E-33	1.0617E-32
3-Hydroxybutan-2-one (acetoin)	54.18 ± 11.93	112.94 ± 36.74	1.18	1.1600E-21	3.9875E-21
Ethyl benzoate	275.74 ± 35.47	565.84 ± 141.54	1.18	5.2075E-30	2.2032E-29
unidentified-10	1755.89 ± 296.36	1086.99 ± 614.21	1.08	7.8699E-12	1.4428E-11
Butan-2-one	3989.17 ± 353.84	2172.00 ± 548.40	1.07	9.4070E-43	1.0348E-41
2-Methylpyrazine-D	188.22 ± 24.63	103.46 ± 19.00	1.03	7.0154E-42	6.4308E-41

Abbreviations: VOC, volatile organic compound; IPM, Imipenem; CSEC, Carbapenem-sensitive *Escherichia coli*; CREC, Carbapenem-resistant *Escherichia coli*; IPM, Imipenem; VIP, variable importance in projection; FDR, false discovery rate; M, monomer; D, dimer; SD, standard deviation.

### 3.7. Potential Application of VOCs in Enzyme Type Identification

To further explore whether VOCs could be used for the recognition of NDM-type carbapenemases, we treated the same 20 CREC strains (each in triplicate, yielding 60 parallel samples per condition) with either IPM alone or IPM combined with DPA under identical experimental conditions. VOC data were collected at the T2 time point for comparative analysis between the two treatment groups. PCA (**Figure 5A**) and PLS-DA (**Figure 5B**) results revealed partial separation between the CREC+IPM and CREC+IPM+DPA groups, although some overlap was observed. Cross-validation yielded *R*^2^ and *Q*^2^ values of 0.86695 and 0.80823, respectively (**Figure S3E**). Additionally, 1000 permutation tests demonstrated no overfitting (*p* < 0.001) (**Figure S3F**).

**Figure 5.**
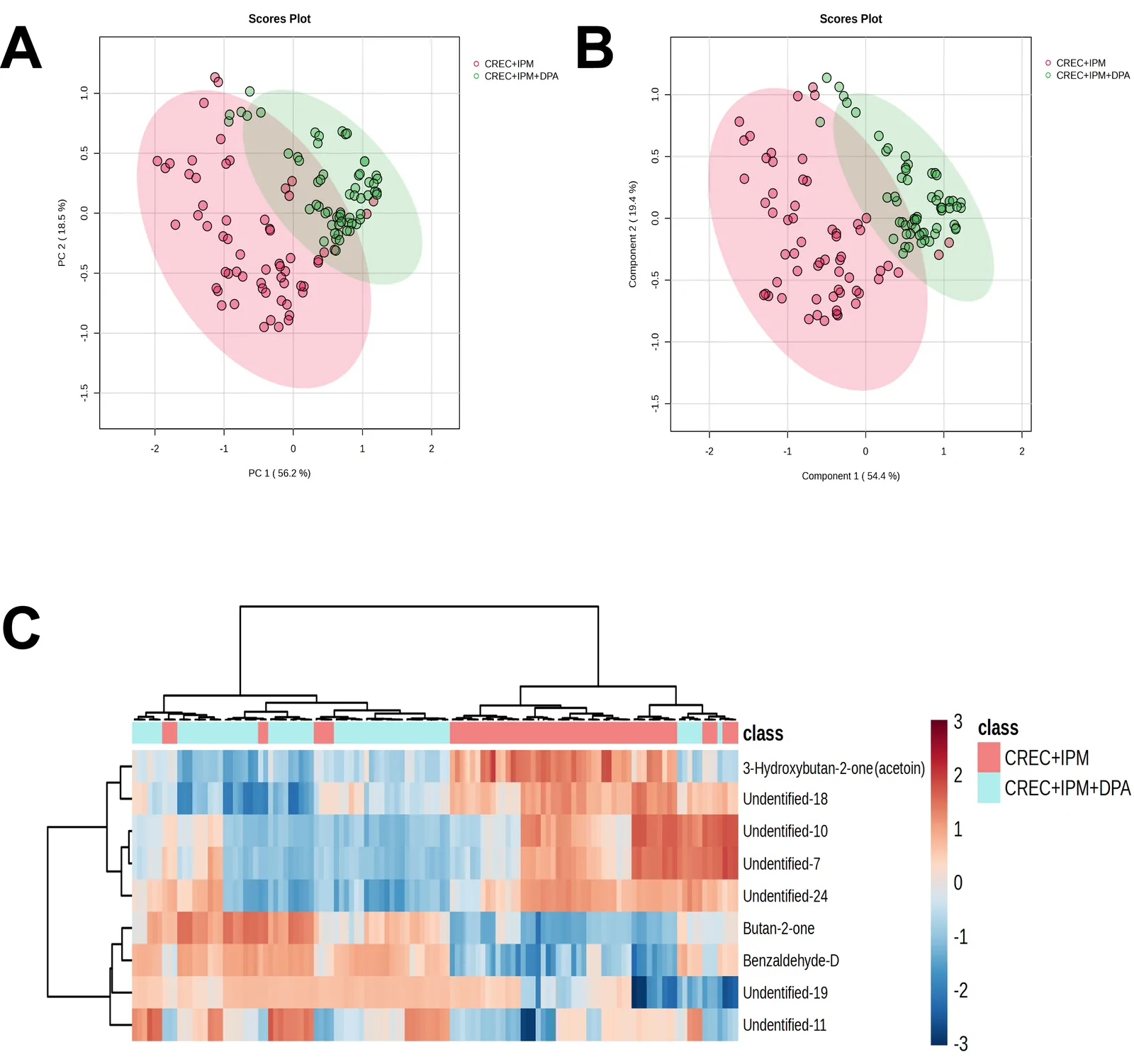
(A) PCA analysis of 60 parallel experiments of enzyme type exploration at time point T2. (B) PLS-DA analysis of 60 parallel experiments of enzyme type exploration at time point T2. (C) The relative abundance of nine different VOCs in the two groups.

The PLS-DA model was used to screen potential VOCs capable of discriminating NDM-type CREC on the basis of VIP values; the results are presented in **Figure S4C**. Nine VOCs with VIP > 1 were identified. After statistical correction, all nine met the selection criteria (VIP > 1 and FDR < 0.05), comprising six unidentified compounds, two ketones, and one aldehyde. These findings are summarized in **Table 4**. Among these nine differentially expressed VOCs, benzaldehyde-D, unidentified-19, unidentified-11, and butan-2-one showed significantly higher levels in the CREC+IPM+DPA group than in the CREC+IPM group, whereas the remaining five exhibited the inverse trend (**Figure S4D**). The relative abundance of these discriminatory VOCs across the two groups is visualized in **Figure 5C**, demonstrating moderate differentiation between the CREC+IPM and CREC+IPM+DPA groups.

**Table 4.** The VOC content of 20 CREC strains added with IPM was compared with that of 20 CREC strains added with IPM+DPA at time point T2.

Label	CREC+IPM (T2) mean ± SD (*n* = 60)	CREC+IPM+DPA (T2) mean ± SD (*n* = 60)	VIP	*p*	FDR
Benzaldehyde-D	735.15 ± 553.25	2523.96 ± 471.47	3.80	7.9373E-38	4.3655E-36
unidentified-18	1639.15 ± 645.16	632.30 ± 390.34	2.74	3.0751E-18	1.6913E-17
unidentified-19	772.59 ± 563.71	1347.99 ± 451.22	2.55	9.7889E-09	2.0707E-08
3-Hydroxybutan-2-one (acetoin)	112.94 ± 36.74	52.06 ± 11.12	1.93	7.5987E-23	1.0448E-21
unidentified-10	1086.99 ± 614.21	542.91 ± 400.71	1.80	7.2525E-08	1.4246E-07
unidentified-11	239.5 ± 94.22	453.35 ± 167.00	1.71	3.2217E-14	9.3261E-14
unidentified-7	41.17 ± 19.83	22.91 ± 15.01	1.58	9.4815E-08	1.7982E-07
unidentified-24	137.82 ± 39.61	83.51 ± 47.10	1.54	3.8005E-10	1.0452E-09
Butan-2-one	2172.00 ± 548.40	3250.42 ± 547.46	1.09	2.8058E-19	2.2045E-18

Abbreviations: VOCs, volatile organic compounds; IPM, Imipenem; DPA, Pyridine-2,6-dicarboxylic acid; SD, standard deviation; CSEC, Carbapenem-sensitive *Escherichia coli*; CREC, Carbapenem-resistant *Escherichia coli*; M, monomer; D, dimer.

## 4. Discussion

*E. coli* remains an important pathogen causing human bacterial infections, and treatment options have become limited because of issues such as antibiotic abuse [16]. Early identification of causative organisms and their potential antibiotic resistance may help reduce the development of bacterial resistance [17] and could even lower patient mortality rates [18, 19]. In this study, GC-IMS technology was used to detect VOCs emitted by *E. coli* cultured in aerobic BacT/Alert® SA bottles at defined time points. The following key findings were obtained: (1) Analysis of VOCs in this broth culture system supports the identification of *E. coli*. (2) The differences in VOCs produced by CSEC and CREC could be amplified under the selection pressure of IPM, which in turn suggested a potential indicator for identifying CREC in the medium of aerobic blood culture flasks. (3) The addition of DPA altered the relative abundance of VOCs in NDM-producing CREC, further indicating the potential of VOCs for identifying carbapenemase types.

The conventional method of microbial identification involves bacterial culture, biochemical testing, and antimicrobial susceptibility testing—a process that typically takes 2–4 days to complete. Given this lengthy timeframe, current microbial identification technologies are too slow to provide timely guidance for antibiotic use during the early stages of patient treatment. The capability of GC-IMS to analyze the headspace of bacterial cultures, combined with its speed and potential for automation, has had a notable impact across various fields, including medicine and environmental health [20, 21]. This technology has shown potential for in vitro monitoring of bacterial growth and strain differentiation. Using GC-IMS, Gallegos et al. identified 2-butanone, 2-pentanone, 2-heptanone, and 3-methyl-1-butanol as key VOCs capable of distinguishing among *Lactobacillus casei*, *Lactobacillus paracasei* subsp. *paracasei*, *Lactococcus lactis* subsp. *lactis*, and *Lactococcus lactis* subsp. *cremoris* [22]. Additionally, researchers have used GC-IMS for real-time monitoring and differentiation of VOCs released by *Fusarium oxysporum* and *Trichoderma atroviride*, further revealing the interaction between these fungi [23]. Taking advantage of the high sensitivity and resolution of GC-IMS, we investigated the volatile metabolic profiles of CSEC and CREC cultured in aerobic blood culture flasks, capturing and analyzing the VOCs released throughout the bacterial growth process. A total of 55 VOCs were detected, of which 30 were tentatively identified. We also investigated the effects of adding antibiotics and carbapenemase inhibitors on the volatile metabolites during bacterial proliferation. Using chemometric and other analytical approaches, we effectively differentiated CREC from CSEC strains. These results indicate that GC-IMS technology has the potential to characterize bacterial sensitivity to antibiotics within a short period, providing timely data to support the optimization of antibiotic treatment protocols by clinicians.

VOCs released by microorganisms exhibit both shared and distinct characteristics [24]. These compounds are generated as primary or secondary metabolites and function in microbial defense or as signaling molecules that facilitate communication between different strains. Although studies have shown that single VOCs can be useful for assessing bacterial growth in vitro [25, 26], the discriminatory power of an individual VOC for distinguishing specific species or strains is limited. Previous research has established that indole is one of the most characteristic VOCs produced by *E. coli* [27–29]. Its biosynthesis involves the tryptophanase (TnaA)-catalyzed deamination of tryptophan to form indole-3-glycerol phosphate, which is subsequently cleaved into indole and pyruvate [30]. Indole functions not only as a signaling molecule in bacterial communication but also enhances *E. coli*’s resistance to environmental stress, such as oxidative stress [31]. However, increasing evidence indicates that multiple bacterial species beyond *E. coli* are also capable of indole production [32].

In this study, indole was not detected. We propose several possible explanations. First, the BacT/ALERT® SA aerobic medium used in this study contains nutrients such as sodium polyanethole sulfonate and mixed amino acids, which may provide amino acids that are more readily utilized than tryptophan. This better utilization may reduce tryptophan catabolism and substantially decrease flux through the indole biosynthetic pathway. Second, the physical properties of indole may contribute to its low detectability. With a molecular weight of 117.15, a melting point of approximately 51–54 ℃, and a boiling point of 253–254 °C, indole exists as a solid or oily liquid at ambient temperatures and exhibits very low volatility. The GC-IMS detection conditions in this study included a headspace temperature of 60 °C (**Table S1**), which may have been insufficient to volatilize indole to detectable levels. Although indole is not exclusive to *E. coli*, its production and relative abundance are typically much higher in this species than in others, consistent with the characteristic odor produced after overnight incubation on blood agar. Thus, *E. coli* still holds a distinctive position in indole biosynthesis.

Among the 13 VOCs tentatively identified as discriminatory between CSEC and CREC, benzaldehyde-D and butan-2-one displayed similar temporal patterns. In the absence of IPM, the relative levels of both VOCs decreased continuously from T0 to T4. However, when IPM was added, these declines occurred only in the CREC group; the CSEC group showed stable levels over time (**Tables S4, S5**). Additionally, Table 4 shows that the relative abundances of benzaldehyde-D and butan-2-one at time point T2 were lower in the CREC+IPM group than in the CREC+IPM+DPA group. These observations suggest that CSEC strains could not withstand IPM-induced selective pressure and ceased metabolic activity at T0. By contrast, the NDM enzyme in CREC facilitates IPM hydrolysis, conferring resistance. When DPA was introduced, it chelated the Zn^2+^ at the NDM active site, preventing IPM hydrolysis; thus, CREC also lost tolerance to IPM [15] and halted metabolism. On the basis of these findings, we infer that benzaldehyde-D and butan-2-one likely function as energy-related metabolites during *E. coli* proliferation. Continuous utilization during growth results in their progressive decline, whereas metabolic cessation leads to stabilization of their levels in the culture medium. In addition, the authors of previous studies have reported the presence of ALDH genes in various bacteria and fungi. ALDHs use nicotinamide adenine dinucleotide (NAD) or nicotinamide adenine dinucleotide phosphate (NADP) as cofactors to oxidize aldehydes, thereby supporting microbial growth and metabolic activity [33–35].

In our previous study, we demonstrated the utility of 3-hydroxybutan-2-one (acetoin) for identifying carbapenem-resistant *Klebsiella pneumoniae* [14, 36]. As a Gram-negative member of the Enterobacteriaceae family, *E. coli* shares several biological characteristics with *K. pneumoniae*, including the ability to survive under aerobic or anaerobic conditions and to generate energy through carbohydrate fermentation. Notably, 3-hydroxybutan-2-one (acetoin) was closely associated with bacterial growth and metabolism in the present study, contributing not only to the differentiation of CSEC from CREC but also to the identification of carbapenemase types. In the absence of IPM, both the CSEC and CREC groups produced higher levels of 3-hydroxybutan-2-one (acetoin) than the blank control group at time point T2 (Table 1) and throughout the monitoring period (**Table S4**). However, after IPM was added, the relative content of 3-hydroxybutan-2-one (acetoin) in the CSEC group remained consistently lower than that in the CREC group across all monitored time points (**Table S5**); this pattern persisted as the number of experimental replicates was increased (Tables 2 and 3). When DPA was introduced to reduce CREC’s resistance to IPM, the relative level of 3-hydroxybutan-2-one (acetoin) following CREC death was markedly lower than that in the surviving CREC group (Table 4). These findings indicate that 3-hydroxybutan-2-one (acetoin) is a valuable marker for distinguishing different Enterobacteriaceae species.

Clinical patients may exhibit substantial variability in the volatile compounds present in their blood due to differences in health status, diet, and lifestyle. For instance, diabetic patients may have elevated ketone bodies; individuals who consume alcohol may have higher alcohol levels; and long-term smokers may inhale nicotine, carbon monoxide, and other volatile substances. Given these potential inter-individual differences, we selected pure cultures of *E. coli* as the research model and used the liquid matrix of clinical blood culture bottles as the medium to evaluate genuine VOC differences between sensitive and drug-resistant strains. This approach represents a major advantage of our study compared with previous work [37]. In our earlier study using tryptic soy broth (TSB) as the culture medium, 36 VOCs produced by *E. coli* were detected using the same analytical workflow, enabling clear discrimination between CSEC and CREC on the basis of differences in relative VOC abundance. In the present study, however, we selected BacT/ALERT® SA as the medium for several reasons. First, although TSB is widely used for bacterial cultivation in laboratory settings, the use of blood culture bottles better reflects clinical conditions. Second, BacT/ALERT FA Plus bottles, which are commonly used in clinical practice, contain adsorptive pellets capable of binding antibiotics or other inhibitory substances present in patient blood samples. Because this study required the use of antibiotics to amplify metabolic differences between CSEC and CREC, the adsorption of IPM by these pellets would interfere with the experiment. By contrast, BacT/ALERT® SA lacks adsorbent pellets, thereby minimizing the risk of false-negative results and improving the reliability and precision of the experimental data. This advantage was a key factor in our selection of this culture system.

Although this study successfully identified carbapenem-sensitive and carbapenem-resistant *E. coli* using GC-IMS and preliminarily explored the potential value of VOCs for identifying NDM-producing CREC, several limitations must be acknowledged. First, the number of experimental strains was small and all were collected from a single center; larger multi-center studies are needed to validate these findings. Second, the effects of blood components were not evaluated, which limits the clinical applicability of the results. Third, this study focused exclusively on NDM-producing CREC. Because CREC strains harboring other carbapenemase types (e.g., KPC or OXA) were not available, expansion of the enzymatic subtype analysis was not feasible. Additionally, because of current limitations of GC-IMS technology and the incompleteness of existing databases, 25 detected compounds could not be identified, restricting our ability to fully characterize microbial metabolites. Finally, the identification of the 30 VOCs reported in this study was based on database matching and should be regarded as tentative in the absence of confirmation using authentic chemical standards. Future work will prioritize validating key differential VOCs through co-injection with reference standards to confirm their identities.

## 5. Conclusions

By integrating GC-IMS technology with chemometric analysis, we successfully identified 13 distinct VOCs produced during the growth of *E. coli* that differentiated CSEC from CREC and preliminarily demonstrated the potential of VOCs to distinguish NDM-producing CREC.

Considering the limitations of this study, future research could be strengthened in several ways. First, because mass spectrometry provides precise molecular weight and fragment ion information that allows structural confirmation of key differential VOCs and systematic analysis of uncharacterized metabolites, future studies could incorporate complementary analytical platforms such as GC-IMS or liquid chromatography–ion mobility spectrometry–mass spectrometry. This strategy would retain the high sensitivity and rapid separation advantages of IMS while leveraging the structural resolution of mass spectrometry, enabling synergistic detection of both volatile and non-volatile metabolites and evaluating their combined diagnostic value for CREC identification. Second, because VOC release is influenced by factors such as temperature and pH, future work should focus on refining culture conditions to continuously validate and optimize key differential VOCs, with the goal of achieving rapid and accurate VOC-based identification within shorter timeframes. Third, building on the clinical isolates collected at our center, collaboration with additional medical institutions will be pursued to expand sample sources, validate the VOC markers identified in this study in a larger and more representative strain cohort, and explore VOCs capable of distinguishing different carbapenemase subtypes. Finally, future studies should extend these analyses to complex biological matrices such as blood to assess their impact on VOC release, detection sensitivity, and specificity. Simulating real clinical scenarios such as bloodstream infections enables the stability and detectability of VOC biomarkers in these matrices to be evaluated, facilitating the transition from in vitro models to direct detection in clinical samples and enhancing the practical utility of this technology.

## Supplementary Material

Supplementary Figures S1-S4

Supplementary Tables S1-S5

## Data Availability

All datasets used during this study were obtained from the Clinical Laboratory of the Second Affiliated Hospital of Nanchang University; for access to datasets under reasonable conditions for the purpose, please contact the corresponding author.

## Abbreviations

ALDH: Aldehyde dehydrogenase
CDC: Centers for Disease Control and Prevention
CHINET: China Antimicrobial Surveillance Network
CLSI: Clinical Laboratory Standards Institute
CRE: Carbapenem-resistant Enterobacteriaceae
CREC: Carbapenem-resistant *Escherichia coli*
CSEC: Carbapenem-sensitive *Escherichia coli*
DNA: Deoxyribonucleic acid
DPA: Pyridine-2,6-dicarboxylic acid
Dt: Drift time
eCIM: EDTA carbapenem inactivation method
E. coli: *Escherichia coli*
EDTA: Ethylenediaminetetraacetic acid
FDR: False discovery rate
GC: Gas chromatography
GC-IMS: Gas chromatography-ion mobility spectrometry
GC-MS: Gas chromatography-mass spectrometry
IMS: Ion mobility spectrometry
IPM: Imipenem
MALDI-TOF/MS: Matrix-assisted laser desorption/ionization time-of-flight mass spectrometry
mCIM: Modified carbapenem inactivation method
MIC: Minimum inhibitory concentration
MODS: Multiple organ dysfunction syndrome
mVOCs: Microbial volatile organic compounds
NAD: Nicotinamide adenine dinucleotide
NADP: Nicotinamide adenine dinucleotide phosphate
NDM: New Delhi metallo-beta-lactamase
NGS: Next-generation sequencing
NIST: National Institute of Standards and Technology
OD: Optical density
PCA: Principal component analysis
PCR: Polymerase chain reaction
PLS-DA: Partial least squares discriminant analysis
RI: Retention index
TnaA: Tryptophanase
TSB: Tryptic soy broth
VIP: Variable importance in projection
VOCs: Volatile organic compounds
WHO: World Health Organization

## CRediT Author Statement

All authors have read and agreed to the published version of the manuscript.

## Supplementary Material

Supplementary Figures S1-S4 and Tables S1-S5. Available at https://doi.org/10.21627/ya61wk37
