# Supplementary material for: GC-IMS Combined with Chemometrics Analysis of Volatile Organic Compounds Facilitates CREC Identification
Source: J Mass Spectrom Adv Clin Lab. 2026 Jul 28;39(1):ya61wk37. doi: 10.21627/ya61wk37 (PMC13750510; doi:10.21627/ya61wk37)
Supplement: Supplementary file 1 [file jmsacl-39-1-ya61wk37-s001.docx]

Supplemental Material : Figures S1-S4

**GC-IMS Combined with Chemometrics Analysis of Volatile Organic Compounds Facilitates CREC Identification**

**Yunwei Zheng, Fuxing Li, Chuwen Zhao, Hong Wang, Shumin Gu, Junqi Zhu, Liuliu Liao, Longhua Hu**

**Figure S1.** Flow chart of the experiment.

**Figure S2.** The schematic diagram of the GC-IMS detection.

**Figure S3.** (A) Results of cross-validation of identification model for blank bottles+IPM, CSEC+IPM and CREC+IPM. (B) Permutation test results of the identification model for blank bottles+IPM, CSEC+IPM and CREC+IPM. (C) Results of cross-validation of identification model for 60 parallel experiments at T2 time point. (D) Permutation test results of the identification model for 60 parallel experiments at T2 time point. (E) Results of cross-validation of identification model for 60 parallel experiments of enzyme type exploration at T2 time point. (F) Permutation test results of the identification model for 60 parallel experiments of enzyme type exploration at T2 time point.

**Figure S4.** (A) Figure of VIP values (VIP > 1) for distinguishing differential VOCs between CSEC+IPM and CREC+IPM. (B) Relative change trend of 13 different VOCs (CSEC+IPM group as reference). (C) Figure of VIP values (VIP > 1) for distinguishing differential VOCs between CREC+IPM and CREC+IPM+DPA. (D) Relative change trend of 9 different VOCs (CREC+IPM group as reference).
